# Longitudinal influence of alcohol and marijuana use on academic performance in college students

**DOI:** 10.1371/journal.pone.0172213

**Published:** 2017-03-08

**Authors:** Shashwath A. Meda, Ralitza V. Gueorguieva, Brian Pittman, Rivkah R. Rosen, Farah Aslanzadeh, Howard Tennen, Samantha Leen, Keith Hawkins, Sarah Raskin, Rebecca M. Wood, Carol S. Austad, Alecia Dager, Carolyn Fallahi, Godfrey D. Pearlson

**Affiliations:** 1 Olin Neuropsychiatry Research Center, Hartford HealthCare Corporation, Hartford, Connecticut, United States of America; 2 Department of Biostatistics, Yale School of Public Health, New Haven, Connecticut, United States of America; 3 Department of Psychiatry, Yale University, New Haven, Connecticut, United States of America; 4 Department of Psychology and Neurosciences, Trinity College, Hartford, Connecticut, United States of America; 5 Department of Community Medicine, University of Connecticut School of Medicine, Farmington, Connecticut, United States of America; 6 Department of Psychology, Central Connecticut State University, New Britain, Connecticut, United States of America; 7 Department of Neurobiology, Yale University, New Haven, Connecticut, United States of America; Centre for Addiction and Mental Health, CANADA

## Abstract

**Background:**

Alcohol and marijuana are the two most abused substances in US colleges. However, research on the combined influence (cross sectional or longitudinal) of these substances on academic performance is currently scant.

**Methods:**

Data were derived from the longitudinal 2-year Brain and Alcohol Research in College Students (BARCS) study including 1142 freshman students who completed monthly marijuana use and alcohol consumption surveys. Subjects were classified into data-driven groups based on their alcohol and marijuana consumption. A linear mixed-model (LMM) was employed using this grouping factor to predict grade point average (GPA), adjusted for a variety of socio-demographic and clinical factors.

**Results:**

Three data-driven clusters emerged: 1) No/low users of both, 2) medium-high alcohol/no-low marijuana, and 3) medium-high users of both substances. Individual cluster derivations between consecutive semesters remained stable. No significant interaction between clusters and semester (time) was noted. Post-hoc analysis suggest that at the outset, compared to sober peers, students using moderate to high levels of alcohol and low marijuana demonstrate lower GPAs, but this difference becomes non-significant over time. In contrast, students consuming both substances at moderate-to-high levels score significantly lower at both the outset and across the 2-year investigation period. Our follow-up analysis also indicate that when students curtailed their substance use over time they had significantly higher academic GPA compared to those who remained stable in their substance use patterns over the two year period.

**Conclusions:**

Overall, our study validates and extends the current literature by providing important implications of concurrent alcohol and marijuana use on academic achievement in college.

## Introduction

Substance abuse (including alcohol) by students is prevalent at U.S. colleges and universities. About 4 of 5 college students drink alcohol and among these, half binge drink [1–3]. Following alcohol, marijuana (MJ) is the most frequent substance of choice among college students [4]. National surveys of individuals aged 18–25 indicate 52% report lifetime use, 31% report usage within the prior twelve months and 21% within the past month [1–3]. Further, 58% of alcohol drinking adolescents report using alcohol and marijuana simultaneously [5].

Recent research indicates that both alcohol and marijuana tend to alter brain structure/function, and are associated with impaired decision making, memory and impulsivity in young adults and adolescents [6–12]. Evidence suggests that adolescents who drink alcohol and also smoke tobacco cigarettes/marijuana are more likely to manifest alcohol and other substance use disorders as young adults compared to those who delay using these substances [13]. More recent evidence also points to the concomitant use of both alcohol and marijuana being associated with abnormal neural tissue development in young adults [14] and worse critical skills such as complex attention, memory, processing speed and visuospatial functioning in adolescents [15]. Despite these documented ill-effects of alcohol and marijuana there has been little change in substance use trends among college students in the past decade [4].

Substance use also has a larger adverse effect on cognitive functions when initiated earlier in life [7, 16, 17]. Early-onset marijuana users demonstrate poorer attention, cognitive inhibition and abstract reasoning, all of which are critical skills needed to function and succeed in a college environment [17]. A large longitudinal study confirmed that adolescent-onset marijuana users showed the largest full scale IQ drop between childhood and adulthood. Moreover, adolescent subjects who used marijuana regularly never reached their predicted IQ levels, even after sustained abstinence [18]. This study also documented that persistent cannabis use was associated with neuropsychological decline across broad functional domains, thus highlighting cannabis’ detrimental long term effects.

Relative to the larger costs to society raised by substance use, academic costs might appear to be minimal. However the impact on academic achievement due to substance use may meaningfully influence long-term student success. Most academic performance research with college students has focused on examining alcohol and marijuana effects separately, with little examination of their combined effects. Because substance use patterns tend to cluster among students, it is important to recognize that their influence on academic outcomes may have separable additive or synergistic effects. Substance use has been directly related to a variety of academic problems; substance-using college students skip more classes, spend less time studying, show decreased motivation and have disrupted sleep patterns [19–24]. Arria et al. performed a large scale longitudinal study which indicated that college students who used marijuana very frequently during all four years were twice as likely as minimal users to experience discontinuous enrollment [20]. These intermediary processes ultimately cascade into more short- and long- term issues such as dropping out of college, losing potential in-college work opportunities, delayed graduation and unemployment [20, 25].

It is important to note that alcohol and marijuana are not the only factors that contribute to poor academic achievement. A variety of other mediating and/or comorbid factors such as socioeconomic status, mental health (anxiety and depression in particular), cultural and family values, stress handling capability, peer pressure, intelligence quotient (IQ), tobacco smoking etc can play a major role in determining academic performance in college students [19, 22, 26–29].

The aim of the current study was therefore to examine the effects of concurrent alcohol and marijuana use on academic performance in a large sample of college students over a two year period. We hypothesized that a) heavy users of either substance would have lower grades than minimal users of both substances and b) students who consumed both alcohol and marijuana heavily would experience a larger decline in GPA over time.

## Methods and materials

A convenience sample of first-year students (age range 18–23 years) was recruited from two local colleges with diverse populations through e-mail, flyers and classroom visits to solicit participation in the Brain and Alcohol Research in College Students (BARCS) study [30]. The recruitment captured greater than 95% of possible participants. Given no minors were recruited; consent was obtained only from subjects and not their parents. All subjects provided written informed consent, including consent to obtain their GPAs each semester of the study. The study was approved by institutional review boards at Central Connecticut State University (CCSU), Trinity College, Hartford Hospital and Yale University. As part of the ongoing study, participants completed online demographic, clinical and substance use surveys every month for two years excluding summer months.

### Variables acquired longitudinally

#### Grade Point Average (GPA)

Individual GPA data (up to 2 decimal places) were collected for each semester from college records.

#### Alcohol use

Two different measures of alcohol use were recorded for each subject on the monthly substance use questionnaires a) the number of days an individual consumed alcohol in the past 30 days and b) the number of drinks he/she had on each occasion.

#### Marijuana use

Students reported their monthly marijuana use in a scaled manner, ranging from 1–6. 1 was defined as having never used marijuana in the past 30 days, 2—having used 1–2 times, 3—having used 3–5 times, 4 –having used 6–9 times, 5 -having used 10–19 times and 6 having used 20+ times in the past month.

### Variables acquired only at semester 1

#### Scholastic Aptitude Test (SAT)

Individual SAT scores for math, verbal and writing were obtained from college records. SAT scores were explicitly included as they have been previously demonstrated to be good predictors of intelligence quotient (IQ) [31].

#### Socio-Economic Status (SES)

SES was calculated using Hollingshead’s four-factor index at the initial visit [32]. Occupational and educational scores were calculated for both parents and then summed to compute a total SES score. Although the direct impact of SES on academic achievement is debatable, we wanted to adjust for any overlapping variance it shared with substance abuse in our current study.

#### Tobacco (Cigarette) smoking

Data on cigarette smoking status was used as a covariate in the current study as it has been shown to be linked with adverse health outcomes in college students [33], which might be an important mediator for academic achievement. Smoking status was assessed using the Fagerstrom test for nicotine dependence [34]. Individual responses from item 4 from the Fagerstrom (How many cigarettes do you smoke per day) was used to identify cigarette-smokers and non-smokers, converted into a binary yes/no format and employed for further analyses.

#### Family History for Alcoholism (FHA)

Students were asked to self-report if they had at least one affected first-degree relative with alcoholism. The above ‘yes/no’ answer was binary coded and used for further analysis. Although studies examining FHA abuse among college students have been largely conflicting, there has been evidence showing FHA is associated with increased levels of alcohol use [28]. We therefore included the above variable as a means to control for such effects in the current analysis.

Lastly, given that anxiety disorders [29, 35, 36] and depression [26, 29] have a direct impact on academic success and often co-exist with substance abuse [37], we administered the mental health questionnaires below at study entry (semester 1) to capture and control such confounding aspects in analysis.

#### State Trait Anxiety Index (STAI)

Students completed the trait sections of the STAI questionnaire [38] at study entry. A total score was computed for each student by summing all these values.

#### Beck Depression Inventory (BDI)

Students were administered the BDI to assess depressive symptoms at study entry [39], all items from the inventory were summed to yield a total score.

### Pre-Processing

Before conducting statistical analysis, alcohol use quantity and frequency were multiplied for each subject to derive total counts of alcoholic drinks consumed per month. Similarly, marijuana scale measures were transformed to continuous measures by taking their midpoint values for each monthly survey input. For example if a student reported consuming marijuana between 6–9 times in the past month, this was converted to a value of 7.5 (midpoint of 6–9). Given that our outcome variable (GPA) was based on a semester basis, we therefore averaged substance use across all months in a semester to derive use measures for that semester. Semester-wise substance use data were then log-transformed and used for further analysis.

Given that the MJ and alcohol use data were highly correlated and skewed (see S1 Fig) we chose to cluster substance use data into groups rather than retaining them as continuous independent predictors in the model in order to estimate valid and non-biased coefficients. A 2-step cluster classifying algorithm was implemented in SPSS based on both alcohol and marijuana consumption measures as input to estimate the underlying data clusters in each semester separately [40, 41]. This clustering algorithm as implemented in SPSS, first constructed a cluster feature tree by placing the first case at the root of the tree in a leaf node, then each successive case was added to an existing node or forms a new node based on its similarity to existing nodes using a distance measure as the similarity criterion. Finally, Individuals were grouped into data-driven clusters using an agglomerative clustering algorithm [39]. These data driven clusters were deemed time-varying and depending on each subjects’ combined substance use patterns for that particular semester, individuals could potentially be categorized under a different cluster membership across each of the four semesters. Akaike’s Information Criterion (AIC) was used to select the number of optimal data clusters across each semester. Resulting cluster assignments of individuals were then tested for their longitudinal inter-reliability using Cohen’s kappa statistic [42].

### Statistical analysis

**Fig 1:**
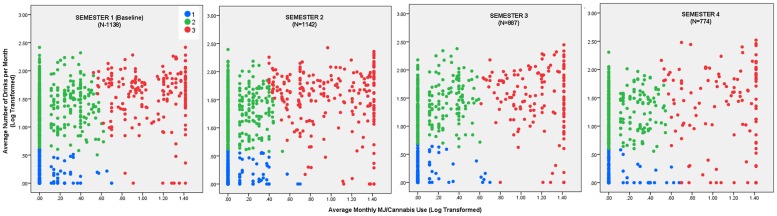
Substance use patterns. Scatter plots of alcohol and marijuana usage among students across semesters.

A longitudinal linear mixed model (LMM) was implemented to assess whether cluster membership predicted changes in GPA across the four semesters. In leiu of missing data the mixed effects approach is advantageous in a few ways over a conventional repeated measures ANOVA model a) it uses all available data on each subject despite missing data by using a maximum likelihood estimation process b) allows greater flexibility in modeling the correlation structure of repeated measures data [43] and c) it relaxes the randomness constraints of the missing data. In the current study, we first considered a full model which included main effects of cluster membership (within-subject, time-varying), sex, semester (within-subject), and the following covariates (captured at semester 1): age, SAT (math, verbal and writing) scores, tobacco smoking status, FHA status, SES, anxiety- and depression-related scores. All interactions between categorical predictors (i.e. sex, semester and cluster) were included in the full model. For parsimony, non-significant interactions, except for the cluster*semester term which interrogated GPA relationship with clusters over time, were dropped in the final model. Post-hoc analyses, including simple cluster effects within each semester, were performed and adjusted for Type I error using the Sidak method. In the above models, the correlation between repeated observations within each subject was modeled by including random subject effects and the best-fitting variance-covariance structure across time. The best fitting model was based on the smallest Akaike information criteria (AIC) [41]. As a secondary analysis, we fit a model including only the main and interactive effects of cluster and semester to examine the overall effects of covariates on the result. All statistical analyses were performed using SPSS v21 (http://www-01.ibm.com/software/analytics/spss/). A total of 1142 subjects were included in the final model; however the number of subjects with fully available data varied for each semester (see Fig 1). Differential data missingness across substance use clusters for semesters 2–4 in relation to semester 1 cluster assignment was assessed using a chi-square test.

In order to get a broad sense of how change in cluster membership (substance use patterns) over the two year period impacted academic GPA we conducted a secondary pseudo-trajectory analysis. In order to do this, each subjects’ data was coded into one of three groups in terms of their substance use as follows a) stable—those who remained in the same cluster at the start and end of the study b) declining—those who moved from a better to worse cluster (cluster 1 to 3, cluster 2 to 3 or cluster 1 to 2) and c) improving—those who moved from a worse to a better cluster (cluster 3 to 2, cluster 3 to 1 and cluster 2 to 1). For simplicity, we only used the starting and the ending semester’s information to decide the above transition groups. Given that there were missing data, the ending semesters were not the same for all subjects. We then performed a LMM similar to our primary analysis, however in this case we replaced the cluster membership predictor that we previously used with the newly coined cluster (group) transition variable and also included semester 1 cluster membership as an additional control variable.

## Results

For subjects in the current study, there were no missing data for alcohol use variables across the four semesters (among subjects that provided data). However, the refusal and/or missing rates for marijuana data were as follows: semester 1: 0.4%, semester 2: 0%, semester 3: 22.3% and semester 4: 32.2% which therefore resulted in 22% of students in the 3^rd^ semester and 32% of students in the 4^th^ semester missing a cluster assignment. S1 Table provides details regarding frequency and missingness of all variables included in the LMM.

### Substance use cluster (group) identification

Three data-driven clusters were identified individually for each of the four semesters. Based on substance use distribution, clusters were labeled as: No-low use of both substances (cluster 1), medium-high alcohol/No-low marijuana (cluster 2), and medium-high use of both substances (cluster 3; see Fig 1). The Silhouette measure of cohesion and separation for the data clusters was 0.7. The silhouette measure represents how close each point in one cluster is to points in the neighboring clusters and thus provides a way to assess parameters such as number of clusters. Silhouette coefficients (as these values are referred to as) near +1 indicates that the sample is far away from the neighboring clusters. A value of 0 indicates that the sample is on or very close to the decision boundary between two neighboring clusters and negative values indicates that those samples might have been assigned to the wrong cluster. In our study a value of 0.7 would refer to a good-very good separation of data. Demographics and clinical characteristics of the clusters at baseline (first semester) are reported in Table 1. Scatter plots of alcohol/marijuana consumption for clusters across semesters are also shown in Fig 1.

**Table 1:** Sample demographics. Demographics and clinical characteristics of study participants at semester 1.

	Cluster 1 No-Low MJ&Alcohol		Cluster 2 Medium-High Alcohol/No-low MJ		Cluster 3 Medium-High Alcohol&MJ		Statistic	
Baseline demographics/clinical characteristics	(N = 487)		(N = 463)		(N = 188)			
	**Mean**	**SD**	**Mean**	**SD**	**Mean**	**SD**	**F**	**p-value**
**Age**	18.32	0.91	18.3	0.73	18.3	0.63	0.13	0.882
**Scholastic Achievement Test (SAT) Math**	541.05	89.52	554.97	90.67	554.23	84.78	3.02	0.05
**SAT Verbal**	530.63	91.04	541.56	89.33	541.24	76.95	1.92	0.146
**SAT Writing**	534.4	90.45	553.75	92.03	544.82	83.87	4.98	0.007
**Grade Point Average (GPA)**	3.1	0.67	3.03	0.64	2.66	0.83	27.75	<0.0001
**Parental Socioeconomic Status (SES)**	12.55	7.04	10.22	5.46	10.24	5.75	19.29	<0.0001
**State-Trait Anxiety Inventory (STAI)**	40.13	9.86	39.23	10.08	41.45	10.69	3.24	0.04
**Beck Depression Inventory (BDI)**	3.32	4.44	3.12	4.43	4.24	5.06	4.12	0.02
**Number of Monthly Drinks**	0.4	0.75	29.29	32.31	54.54	42.69	311.17	<0.0001
**Monthly Marijuana Usage (Frequency)**	0.08	0.39	0.42	0.71	13.54	8.13	1242.52	<0.0001
	**N(%)**		**N(%)**		**N(%)**		**Chi-Square**	**p-value**
**Gender**								
**Male**	186		173		101		15.49	<0.0001
	(38.2)		(37.3)		(53.7)			
**Female**	299		286		87			
	(61.8)		(62.7)		(46.3)			
**Cigarette Smoker**								
**No**	459		411		147		45.28	<0.0001
	(94.2)		(88.8)		-78.2			
**Yes**	19		42		38			
	(3.9)		(9)		(20.2)			
**Missing**	9		10		3			
	(1.9)		(2.2)		(1.6)			
**Family History for Alcoholism**								
**Negative**	378		365		139		0.4	0.397
	(77.6)		(78.8)		(73.9)			
**Positive**	109		98		49			
	(22.4)		(21.2)		(26.1)			

Data missingness in semester 3 and 4 (in relation to semester 1 cluster assignment) was significantly different across the three substance use clusters. For semester 3, missingness statistics (chi-square 19.81; p<0.001) were as follows: 17.3% for cluster 1, 23.3% for cluster 2 and 32.9% of cluster 3. Similarly missingness for semester 4 (chi-square 36.83; p<0.001) data was as follows: 26.1% for cluster 1, 31.3% for cluster 2 and 50% of cluster 3.

Cluster coherence among individuals was fairly stable when assessed in relation to baseline (SEM1→SEM2: κ = 0.64; p<0.001, SEM1→SEM3: κ = 0.70; p<0.001, SEM1→SEM4: κ = 0.67; p<0.001). These kappa values suggested good-very good agreement of designated cluster assignment of subjects across semesters compared to semester 1 values. Comparable cluster coherence emerged when assessed on a semester-to-semester basis.

### Linear Mixed-Model (LMM) analysis

**Fig 2:**
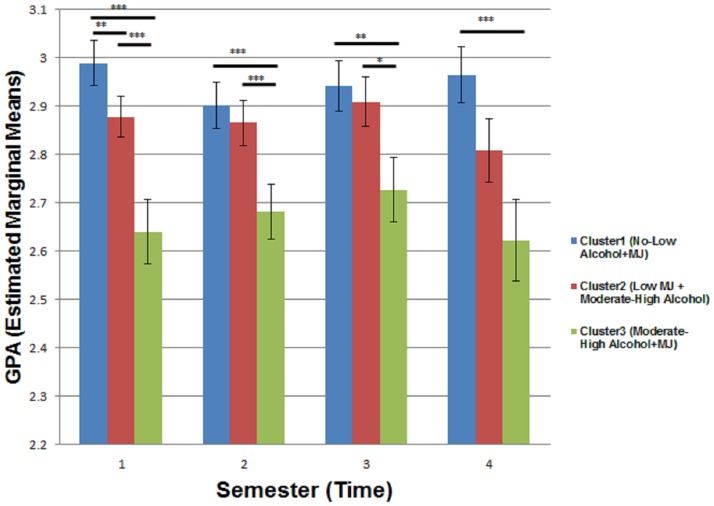
Linear mixed model plot. Bar plot of GPA (estimated marginal means) across semesters by cluster. Post-hoc between-group analysis (using a sidak correction for multiple comparisons) is also indicated. Between-cluster, post-hoc significances are marked as follows: ***p<0.001, **p<0.005,*p<0.01. Note: Error bars represent standard error of mean.

**Fig 3:**
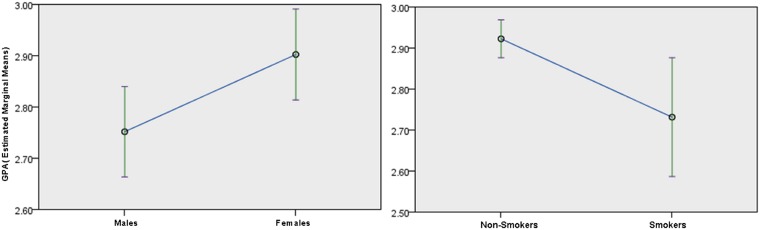
Mean plots of GPA (estimated marginal means) across gender and smoker groups. Error bars across means represent 95% confidence interval limits.

As expected, the main effect of cluster membership (F (2,2267.8) = 20.6; p<0.001) was a significant predictor of academic performance after controlling for clinical and demographic variables. Post-hoc analyses showed that compared to cluster 1 students (who consumed little-no alcohol/marijuana); those who consumed moderate-high level of alcohol (cluster 2) obtained lower average GPAs (averaged across semester). Further, students in cluster 3 (who consumed medium-high quantities of both substances) obtained lower grades compared to both cluster 1 and cluster 2 students. No significant higher-order interactions were observed. Although not significant, per our initial hypothesis, the cluster*semester term was retained. Simple effects of cluster within each semester (cluster*semester) revealed differences in GPA between clusters 1 and 2 were significant only at semester 1 (See Fig 2). However, significantly reduced GPA was noted in cluster 3 subjects compared to cluster 1 or 2 students across each of the four semesters. In addition to cluster membership, tobacco smokers (shown in Fig 3) had significantly lower GPA than non-smokers (F(1,873.2) = 4.16; p<0.05), males had significantly lower GPA compared to females (F(1,844.6) = 12.26; p<0.001), SAT scores positively predicted GPA (math (F(1,822.27) = 22.27; p<0.0001), writing (F(1,830.16) = 16.24; p<0.001)) and BDI was negatively associated with GPA (F(1,851.80) = 4.51; p = 0.03)). LMM evaluated without any covariates yielded exactly the same conclusions as above for the main effect of cluster, semester and their interaction.

**Fig 4:**
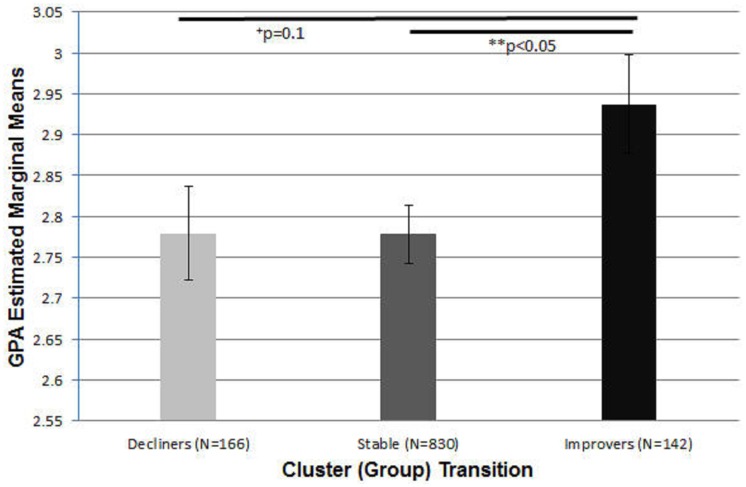
Bar plot of GPA (estimated marginal means) across cluster (group) transitioners from our pseudo trajectory analysis. Post-hoc between-group analysis (using a Sidak correction for multiple comparisons) is also indicated. The Improver group represents students that went from a worse substance use cluster to a better one over the period of the study. Decliners were the opposite of improvers. Stable group participants started and ended the study in the same substance use cluster. Note: Error bars represent standard error of mean.

Our secondary pseudo-trajectory analysis indicated a significant main effect of the cluster transition predictor. Post-hoc effects revealed that the improving group had significantly higher academic GPA compared to the stable group (see Fig 4). All other post-hoc comparisons were non-significant for this predictor. In addition we also noted semester 1 cluster membership, SAT math, SAT writing, sex and smoking status all to be significantly associated with academic GPA as shown in Table 2 below.

**Table 2:** Fixed effects from the linear mixed model conducting the pseudo trajectory analysis.

Source	F	Sig.
Intercept	2.282	.131
Smoking Status	8.007	.005
Family History	2.387	.123
Sex	14.294	<.0001
Cluster Transition	3.617	.027
Semester 1 Cluster	22.838	<.0001
Semester	1.029	.379
Age	.165	.685
SAT_Math	22.787	<.0001
SAT_Verbal	.472	.492
SAT_Writing	15.302	<.0001
STAI	.114	.736
BDI	3.510	.061
Parental SES	3.381	.066
Semester1 Cluster by Semester	.816	.557
Cluster Transition by Semester	.821	.553

## Discussion

To our knowledge this is the first large-scale study to simultaneously integrate the effects of alcohol and marijuana use on college GPA using a longitudinal statistical model. Given the challenges presented by comorbid and collinear use of both alcohol and marijuana in college students, we performed a data-driven categorization of cumulative substance use and then used a LMM approach to evaluate the longitudinal effects of both alcohol and marijuana use on individual college GPA in a cohort of 1000+ college students. However, it is important to note that our study design or analysis was not geared to examine causation. Given the analysis presented in the paper, all we can infer is that academic performance and substance use was significantly associated. Our data-driven analysis revealed that roughly 40% of the students did not use, or consumed very light amounts of alcohol and MJ. Students who consumed moderate-to-large amounts of alcohol but only small-no MJ comprised another 40%, and the rest of the sample was heavy users of both substances. We also found almost no individuals with minimal alcohol and heavy MJ use. This is interesting given the popular belief that MJ-only users are much more common than individuals who use both substances together. However, the data driven patterns of substance use clusters we found was largely consistent with past reported data in college students [22].

The substance-use clusters identified across individuals in our study were relatively stable across semesters. Thus, heavy substance users during freshman year were more likely to maintain the same use pattern throughout the 2 study years. As hypothesized, even after controlling for a variety of demographic and clinical characteristics, cluster 3 students (who were moderate-heavy users of both substances) had poorer grades (from study outset to completion) than their peers who consumed little or no alcohol/marijuana. Overall, cluster 2 students (moderate-high alcohol + low-or-no marijuana) had lower GPA’s compared to their non/low substance use peers. However, contrary to our hypothesis we found no cluster by semester interaction, which suggests that the overall relationship between cluster and GPA is the same across each semester (as seen in Fig 2). Despite a non-significant interaction term, we looked at post-hoc simple effects to better interpret the data. These analyses revealed that the difference in GPA between cluster 1 and 2 diminished to trend-level significance during semester 2 and was non-significant for semesters 3 and 4. One possible explanation is that students consuming moderate-to-high levels of alcohol and only low levels or no marijuana (cluster 2), adapt over time, develop tolerance to substance effects and/or regulate their behavior to eventually catch up academically with their sober peers. Alternatively, it is possible that cluster 2 students chose to switch to less academically challenging courses. We were not able to distinguish among these possibilities.

The above was however not true for students who heavily used both MJ and alcohol (cluster 3). Despite the fact that cluster 3 students had similar SAT scores of academic achievement coming into college, compared to cluster 1 and 2, we noted that by the end of semester 1, they had significantly reduced GPAs. Our results therefore suggest that consuming moderate-to-high levels of both substances were associated with significantly lower GPA, which seemed to follow across subsequent semesters (see Fig 2). However, this level of consumption was not associated with a decline in GPA from semester 1, i.e. when we tracked cluster 3 academic performance over time, there was no significant change in GPA over the two-year period. Although partly consistent with our initial hypothesis, this particular finding was not entirely expected, as we had initially predicted that engaging in heavy substance use of both substances would also result in a faster decline in academic achievement over time (i.e. we would see a significant cluster by semester interaction). One simple possibility is that we had too few time points in the study in later semesters to detect a significant GPA decline over time. Also, larger attrition of subjects across substance use clusters over time could have resulted in a non-significant decline of GPA as well. Finally, it could be possible that heavy substance users are able to tolerate deleterious effects of those substances, due to perhaps a combination of genetic predisposition and adaptive cellular or systemic tolerance, thus preventing them from a continuing decline in academic performance. This hypothesis is supported in-part by evidence from both the animal and human literature. A rodent study demonstrated that exposures to large quantities of alcohol led to functional tolerance independent of environmental influences [44]. In humans, a study investigating alcohol tolerance in offspring of alcoholics and non-alcoholics, found that the former experience alcohol’s pleasurable effects at the start of drinking and are less affected by its subsequent impairing effects during the drinking session, that in turn might contribute to increased drinking behaviors [45, 46]. Similar animal-based evidence for tolerance effects at both the observational and neuronal levels has been noted for heavy marijuana use [47]. It is possible that a large number of students belonging to cluster 3 fit the above pattern and were thus able to maintain their grades over time due to adaptive tolerance to heavy substance use.

A key strength of the current study is its focus on a critical age group, college-attending late adolescents to early adults, when substance use and initiation is prevalent. Animal studies suggest that compared to adults, adolescents may be particularly sensitive and vulnerable to neurotoxic effects of substances including alcohol and marijuana [48–50]. Preclinical studies also report increased cellular changes in adolescents compared to adults associated with exposure to THC (delta-9- tetrahydrocannabinol), a major psychoactive compound in marijuana [48, 49]. Human studies indicate that regular marijuana use starting before age 18 is associated with deficits in attention, visual search, executive functioning and IQ [7, 9, 16–18]. Newer research seems to indicate that persistent cannabis use across four years of high school is associated with lower GPA, SAT scores and higher externalizing symptoms [51]. A recently-published large longitudinal cohort study that utilized trajectory analyses, seems to indicate that students who smoked cannabis (irrespective of use being infrequent, frequent, increasing or decreasing) during college had significantly lower college GPA and other related academic outcomes (even at study onset) [52]. Another study used structural equation modeling to conclude that MJ use during the first semester was directly related to lower GPA in college students. The study also found similar indirect effects of alcohol and other substances on academic outcomes such as GPA and graduation time [53]. Our results both confirm and extend previous reports by suggesting that students who consume moderate-large quantities of both alcohol and marijuana show significantly lowered college academic performance that is seen consistently over a two-year period. It was also encouraging to observe that students who in general were able to moderate or lower substance use patterns while in college over time performed significantly better academically compared to students who remained in the same substance use cluster. Our pseudo-trajectory analysis might therefore provide important clues in terms of how regulating, curtailing and modifying substance use behavior while in college could have a significant impact on academic GPA.

Our results suggest that early intervention to substance abuse in college students might be vital in shaping their academic success over time. Students who use alcohol and MJ (especially MJ) minimally during their incoming semester seem to do better academically over time. This might in-turn lead to significantly better life outcomes upon graduation. It was interesting to note that despite no differences in SAT scores between clusters, we begin to see a dramatic difference in GPA associated with heavy substance use by the end of the first semester. First-year students typically have exaggerated ideas about how much college students drink. Wanting to fit in and being free of parental control for the first time, these students can be led by this misperception into a pattern of heavy drinking that increases their risk of academic failure, serious injury, sexual assault, and even death. As stated by Moos et al 1976, “College health and academic counseling services may benefit from knowledge of individuals at high risk for poor physical health, academic dropout, and alcohol abuse.” This could translate into identification of heavy student alcohol and substance users through improved substance use education and readily available counseling programs, with the aim of reducing substance-associated morbidity. It is typical for freshman students to be subjected to peer pressure of drinking given the somewhat false sense of excessive drinking and substance use culture in colleges. One way to counteract this would be to roll out social norms marketing campaigns that try to convey accurate survey data about student drinking norms. The idea is that once students learn that far fewer students are drinking heavily than they once thought, they will feel less social pressure to drink and, therefore, moderate their alcohol use. In addition to enforcing strict rules and disseminating knowledge on alcohol and substance abuse during social events especially during the freshman year, schools could also take a more proactive role in creating a substance-free environment by promoting alcohol-free events for students and supporting student clubs and organizations that are substance-free and create and promote service learning and volunteer opportunities that are an integral part of the academic curriculum.

As mentioned previously mental illness is an important piece of the picture, given that psychiatric disorders and substance abuse are often co-morbid; we tried to control for these important factors. However, these measures were only collected at study onset, future studies might therefore benefit by collecting history of tobacco use, depression and anxiety measures longitudinally to better account for variability in these potentially important mediating factors. In the current study however, exploratory analyses performed within the LMM framework found no interaction between depression or anxiety scores with cluster definition.

Other strengths of the current study included being among the first to assess simultaneous longitudinal influences of both alcohol and marijuana on academic performance. Further, we controlled for a variety of demographic and clinical variables that might otherwise be comorbid with substance use. The study also had several limitations. We were not able to detect a meaningful group of individuals with low alcohol and moderate-high marijuana use, which would have allowed us to better parse effects of substance use on academic performance. Unfortunately we did not capture a variety of other interesting mediating factors such as “pre-college” drinking or marijuana use patterns, high school GPA, skipping classes, course difficulty or degree of college engagement which could have enabled us to draw better inferences. There was a significant degree of non-reporting of marijuana use in later semesters, in subjects who volunteered other types of data; we do not have a ready explanation for the specific failure to report these substance data. However, given that missingness seemed to increase from cluster 1 to cluster 3 in a step-wise manner, one might speculate that participants who frequently abused substances were less motivated to provide survey data as time progressed. The above missing data was also significantly different across substance use clusters which could have possibly impacted our findings.

## Conclusions

Overall, our study adds to the growing body of adolescent/early adulthood substance use literature by showing that combined alcohol and marijuana effects can pose a threat to college GPA. Although we detected significant differences in academic GPA across different substance use groups, the association between substance use (clusters) and GPA did not change over the 2-year follow up period. Students consuming moderate-heavy alcohol and little-no marijuana seemed to show significantly lower GPA only at study onset. However the impact on GPA in students who consumed moderate-heavy doses of both alcohol and marijuana seemed to be more severe as these students had a significantly lowered GPA at both outset and throughout the 2-year study period, suggesting serious implications towards academic achievement when both substances are prevalently used while attending college. Supplementary results also suggest that moderating or curtailing substance use while in college might help improving academic GPA.

## Supporting information

S1 Table Frequency and missingness of key variables included in the linear mixed model. (DOCX)

S1 Fig Alcohol and marijuana use across each semester. (TIF)
