# Supplementary material for: Longitudinal influence of alcohol and marijuana use on academic performance in college students
Source: PLoS One. 2017 Mar 8;12(3):e0172213. doi: 10.1371/journal.pone.0172213 (PMC5342177; doi:10.1371/journal.pone.0172213)
Supplement: S1 Table — (DOCX) [file pone.0172213.s001.docx]

**Supplementary Table 1:**

| **Variables** | **Cases (N=1142)** | | | |
| --- | --- | --- | --- | --- |
|  | **Valid** | | **Missing** | |
|  | **N** | **Percent** | **N** | **Percent** |
| **GPA Semester 1** | 1138 | 99.6% | 4 | .4% |
| **GPA Semester 2** | 1117 | 97.8% | 25 | 2.2% |
| **GPA Semester 3** | 1036 | 90.7% | 106 | 9.3% |
| **GPA Semester 4** | 986 | 86.3% | 156 | 13.7% |
| **Cluster Definition**  **Semester 1** | 1138 | 99.6% | 4 | .4% |
| **Cluster Definition**  **Semester 2** | 1142 | 100.0% | 0 | 0.0% |
| **Cluster Definition**  **Semester 3** | 887 | 77.7% | 255 | 22.3% |
| **Cluster Definition**  **Semester 4** | 774 | 67.8% | 368 | 32.2% |
| **Sex** | 1135 | 99.4% | 7 | .6% |
| **Age** | 1134 | 99.3% | 8 | .7% |
| **SAT Math** | 1041 | 91.2% | 101 | 8.8% |
| **SAT Verbal** | 1041 | 91.2% | 101 | 8.8% |
| **SAT Writing** | 1038 | 90.9% | 104 | 9.1% |
| **Smoking Status** | 1120 | 98.1% | 22 | 1.9% |
| **STAI** | 1102 | 96.5% | 40 | 3.5% |
| **BDI** | 1142 | 100.0% | 0 | 0.0% |
| **Family History**  **Alcoholism Status** | 1142 | 100.0% | 0 | 0.0% |
| **Parental SES** | 1142 | 100.0% | 0 | 0.0% |
