# Supplementary figures and images for: Longitudinal influence of alcohol and marijuana use on academic performance in college students
Source: PLoS One. 2017 Mar 8;12(3):e0172213. doi: 10.1371/journal.pone.0172213 (PMC5342177; doi:10.1371/journal.pone.0172213)

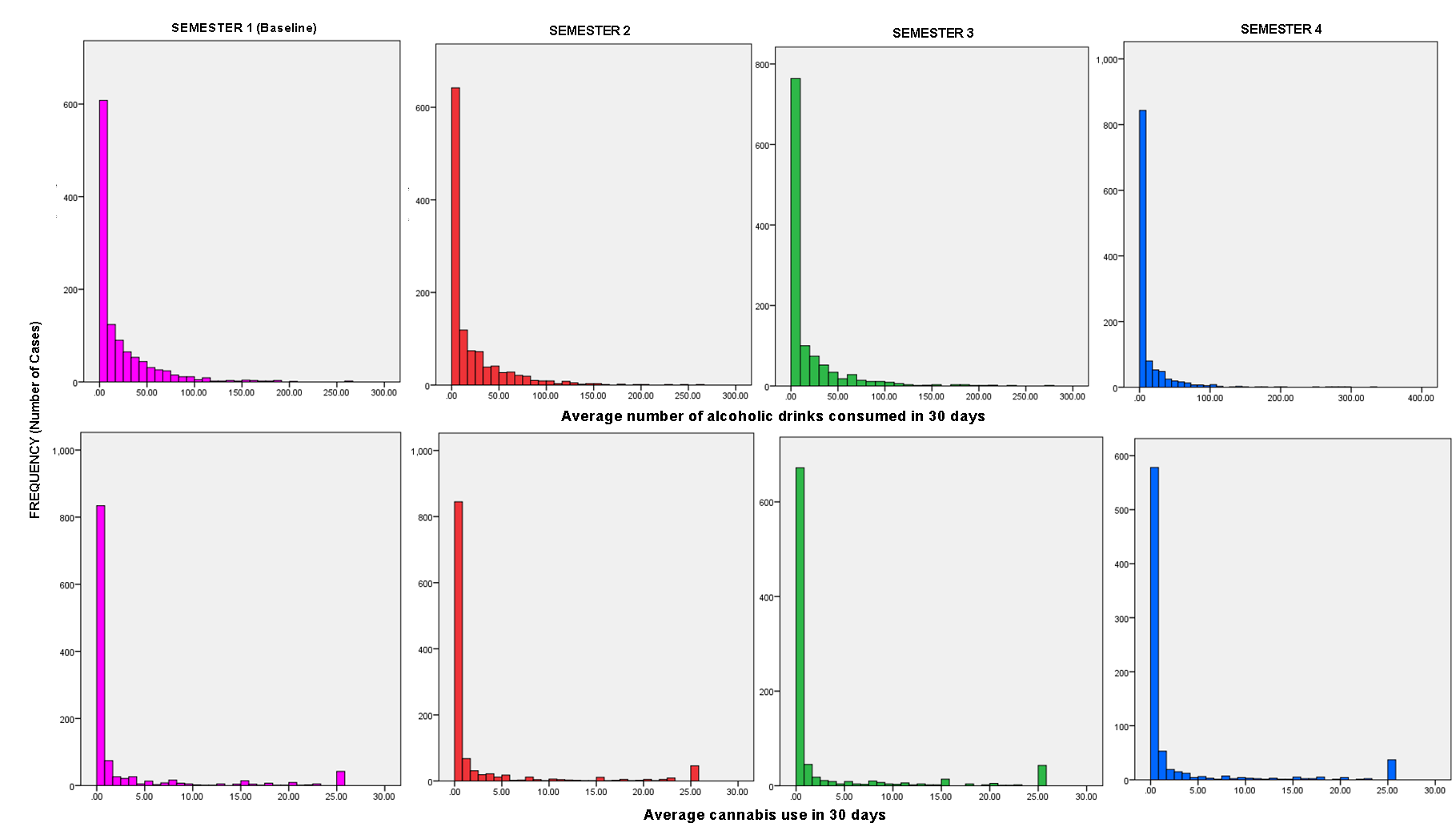

Supplement: S1 Fig — (TIF) [file pone.0172213.s002.tif]
